# Epidemiological Parameters of COVID-19: Case Series Study

**DOI:** 10.2196/19994

**Published:** 2020-10-12

**Authors:** Shujuan Ma, Jiayue Zhang, Minyan Zeng, Qingping Yun, Wei Guo, Yixiang Zheng, Shi Zhao, Maggie H Wang, Zuyao Yang

**Affiliations:** 1 Department of Epidemiology and Health Statistics Xiangya School of Public Health Central South University Changsha China; 2 Peking University Shenzhen Hospital Shenzhen China; 3 Department of Social Medicine and Health Education School of Public Health Peking University Beijing China; 4 Xiangya Hospital Central South University Changsha China; 5 JC School of Public Health and Primary Care The Chinese University of Hong Kong Hong Kong Hong Kong

**Keywords:** coronavirus disease 2019, COVID-19, incubation period, serial interval, basic reproduction number, presymptomatic transmission

## Abstract

**Background:**

The estimates of several key epidemiological parameters of the COVID-19 pandemic are often based on small sample sizes or are inaccurate for various reasons.

**Objective:**

The aim of this study is to obtain more robust estimates of the incubation period, serial interval, frequency of presymptomatic transmission, and basic reproduction number (R_0_) of COVID-19 based on a large case series.

**Methods:**

We systematically retrieved and screened 20,658 reports of laboratory-confirmed COVID-19 cases released by the health authorities of China, Japan, and Singapore. In addition, 9942 publications were retrieved from PubMed and China National Knowledge Infrastructure (CNKI) through April 8, 2020. To be eligible, a report had to contain individual data that allowed for accurate estimation of at least one parameter. Widely used models such as gamma distributions were fitted to the data sets and the results with the best-fitting values were presented.

**Results:**

In total, 1591 cases were included for the final analysis. The mean incubation period (n=687) and mean serial interval (n=1015 pairs) were estimated to be 7.04 (SD 4.27) days and 6.49 (SD 4.90) days, respectively. In 40 cases (5.82%), the incubation period was longer than 14 days. In 32 infector-infectee pairs (3.15%), infectees’ symptom onsets occurred before those of infectors. Presymptomatic transmission occurred in 129 of 296 infector-infectee pairs (43.58%). R_0_ was estimated to be 1.85 (95% CI 1.37-2.60).

**Conclusions:**

This study provides robust estimates of several epidemiological parameters of COVID-19. The findings support the current practice of 14-day quarantine of persons with potential exposure, but also suggest the need for additional measures. Presymptomatic transmission together with the asymptomatic transmission reported by previous studies highlight the importance of adequate testing, strict quarantine, and social distancing.

## Introduction

In December 2019, a novel enveloped ribonucleic acid (RNA) beta-coronavirus, which was later named SARS-CoV-2, was first reported in Wuhan, the capital city of Hubei province, China [1]. COVID-19, the disease caused by SARS-CoV-2, spread across and outside China rapidly, and was declared a pandemic by the World Health Organization on March 11, 2020.

Despite the explosive growth of the number of studies on COVID-19 [2-4], several key epidemiological parameters of the disease remain to be clarified, among which are incubation period and serial interval. The mean or median incubation period and serial interval estimated by previous studies were mostly 4-5 days [5-16]. However, many of the studies included a limited number of cases (around or less than 100) [5,7,9,12-16]. For example, in a study by Zhang et al [16], which included 8579 cases, only 49 cases and 39 pairs could be used for estimating incubation period and serial interval, respectively. On the other hand, some studies might have been limited by the inaccuracy of the original data. For example, if the interval of exposure was long or unclear, determining the exact exposure date would be difficult [6,8,17], which could give rise to error.

Another important parameter of transmission dynamics is the basic reproduction number (R_0_), which is defined as the average number of secondary cases caused by a single infectious individual in a totally susceptible population [18]. As R_0_ is often estimated based on the serial interval [19], the abovementioned issues affecting previous estimates of serial interval might have affected the estimates of R_0_ as well. On the other hand, some studies estimated R_0_ based on the latent period and infectious period [19,20], with latent period approximated by incubation period. This may not be appropriate for COVID-19 as presymptomatic transmission might occur [21,22]. However, few published studies [23] estimated how often and approximately when the disease could be transmitted prior to symptom onset.

This study made use of the large amount of data reported by the health authorities of and outside China, as well as data from studies published as of April 8, 2020, to address the above issues. Specifically, we aimed to obtain more robust estimates of the following epidemiological parameters of COVID-19: (1) incubation period, defined as the time interval between exposure and onset of disease symptoms; (2) serial interval, defined as the duration between symptom onset of an infector (eg, a primary case) and that of an infectee (eg, a secondary case) in a transmission chain, with a negative value meaning that the infectee’s symptoms occurred before the infector’s symptoms; (3) frequency of presymptomatic transmission; and (4) R_0_.

## Methods

### Data Sources

The details of data sources can be found in Multimedia Appendix 1. For China, all provinces, autonomous regions, and municipalities (including mainland China, Hong Kong, Macau, and Taiwan) that had reported cases of COVID-19 were identified according to the daily updates by the National Health Commission of China [24]. Subsequently, the official websites and WeChat accounts (if any) of local governments and health authorities (eg, Municipal Health Commission, Center for Disease Control and Prevention, Department of Health) were checked manually through April 8, 2020, to identify and download the reports on laboratory-confirmed cases of COVID-19. Google and Baidu were searched to identify public media reports written based on official press releases. Chinese words for the following terms were used to perform the search: (“family” OR “household”) AND (“cluster” OR “dinner” OR “party”) AND “infection.”

Other countries reporting COVID-19 cases were identified according to the COVID-19 situation reports published by the World Health Organization [25] and the data were searched through April 8, 2020. For Japan and Singapore, information regarding confirmed cases was retrieved from their respective Ministry of Health. We also searched for individual cases from relevant departments and public media of the United States, the United Kingdom, Canada, and Australia, but failed to find any with details allowing for parameters estimation for this study. Typically, the dates of exposure and symptom onset were lacking (eg, see reference [26]). Owing to the language barrier, we did not do a comprehensive search for other countries.

PubMed was searched to identify relevant publications by using the following terms: “coronavirus,” “2019-nCov,” “SARS-CoV-2,” and “COVID-19.” The China National Knowledge Infrastructure (CNKI) was searched to identify publications in Chinese journals using “novel coronavirus” (“Xin Xing Guan Zhuang Bing Du” in Chinese pinyin), which is the official Chinese name for SARS-CoV-2. Both databases were searched from December 2019 through April 8, 2020. The reference lists of eligible publications were also checked to see if there were other eligible studies not found by previous searches. We also kept an eye on the COVID-19 studies disseminated by public media and the official WeChat accounts of various academic entities in China and those recommended by experts in this field to the authors of this study.

### Definitions and Inclusion Criteria

To be eligible, a report had to contain individual data that allowed for estimation of at least one of the following parameters of laboratory-confirmed cases of COVID-19: incubation period, serial interval, and symptoms-to-transmission time, which was defined as the day of an infectee’s contact with the infector relative to the latter’s symptom onset date, with a negative value meaning that the transmission occurred before the infector developed symptoms (ie, presymptomatic transmission).

To obtain an accurate estimate of incubation period, only the cases with an exposure period spanning 3 days or less were included in the analysis. For those exposed for three continuous days and those exposed on two dates with one day apart (ie, exposed on the first and third days), the second day was uniformly used as the exposure date in estimations. For those exposed for two continuous days, the first day was uniformly used as the exposure date in estimations. This approach ensured the upper limit of error in the estimated incubation period be smaller than 1 day for the cases with a 2- or 3-day exposure, regardless of when exactly (ie, first, second, or third day) the transmission occurred. The actual overall error was bound to be much smaller than 1 day, as most included cases were exposed for only 1 day, which would dilute the error caused by 2- or 3-day exposures.

Serial interval was estimated based on the symptom onset dates of infector-infectee pairs, which were typically from cluster infections. For two cases to qualify as an infector-infectee pair and be included in this study, the following two criteria must both be fulfilled. First, there must be evidence that the presumed infector had been exposed outside the cluster (eg, close contact with a confirmed case, travel history to Hubei, exposure to a person who returned from Hubei) before he/she attended the group activities (eg, family gathering, business conference) that led to cluster infections. Second, in the 14 days prior to symptom onset, the presumed infectee was exposed to the presumed infector only, without other exposure histories.

The estimation of symptoms-to-transmission time also involved determination of exposure date and judgement about transmission chain, hence the above principles applied in estimating incubation period and serial interval were followed as well.

### Screening, Data Extraction, and Quality Control

In total, 6 researchers were involved in data collection. The reports retrieved through the above searches were scrutinized one by one according to the inclusion criteria specified above. The following data were extracted from eligible reports by using a standard extraction form which was pilot tested with the reports from Liaoning province of China: the geographical location concerned, age, sex, type of exposure, first date and period (if applicable) of exposure, date of symptom onset, initial symptoms, and whether the case was from a cluster. For a clustering case, the generation he/she belonged to, the exposure date, and symptom onset date were also recorded. The retrieved reports were split into six parts, with each researcher responsible for one part. For each part, the eligibility of and data extracted from reports were first determined by one researcher and then cross-checked by another three researchers. All uncertainty and disagreements were discussed among the researchers. If no consensus could be reached after discussion, the concerned cases would be excluded to ensure the correctness and accuracy of data. For example, if it could not be determined who was infected through attending a group activity (ie, infectee) and who had been infected before he/she attended the activity (ie, infector), then all clustering cases related to the activity had to be excluded.

### Data Analysis

The basic characteristics of included cases were summarized descriptively. Lognormal, Weibull, and gamma distributions were fitted to the data sets of incubation period and serial interval and the one with the smallest Akaike information criterion (AIC) score was used for the final analysis. For serial interval, which had negative values, shifted distributions were fitted, with the best-fitting values determined by maximum likelihood. The key parameters were estimated by using the maximum likelihood approach. The data on symptoms-to-transmission time was roughly symmetrical according to visual inspection and thus fitted by normal distribution. For each parameter, the range, median, selected percentiles, mean, and standard deviation were estimated. The 95% confidence intervals of mean and standard deviation were estimated by using the bootstrap technique. R_0_ was estimated by the well-studied Euler-Lotka equation [19]: R_0_ = 1/*M*[−*r*|*h*(∙)], where r is the exponential growth rate, *h*(∙) is the estimated distribution of the generation time, and the function *M*[∙] is the Laplace transform (ie, moment generating function known for statisticians) for *h*. As generation time is hard to observe directly, it was approximated by serial interval in this study. Compared with generation time, serial interval is expected to have the same mean but a larger variance due to possibly differing incubation periods of infectors and infectees. The exponential growth rate was obtained directly from a previous study reporting incidence data of the early stage of the epidemic [1], while the other parameters came from this study.

Sensitivity analyses were conducted to examine the robustness of the two parameters involving exposure date. Specifically, the third day for those whose exposure period spanned three days and the second day for those exposed for two continuous days were used as their exposure dates in sensitivity analyses. The difference between the estimates from sensitivity analysis and those from primary analysis represents the largest possible error in the latter. All statistical analyses were conducted with SAS software (Version 9.4, SAS Institute).

### Ethical Statement

No ethical approval was needed as the data used in this study was publicly available, either released by health authorities or retrieved from electronic databases.

## Results

### Overview

**Figure 1:**
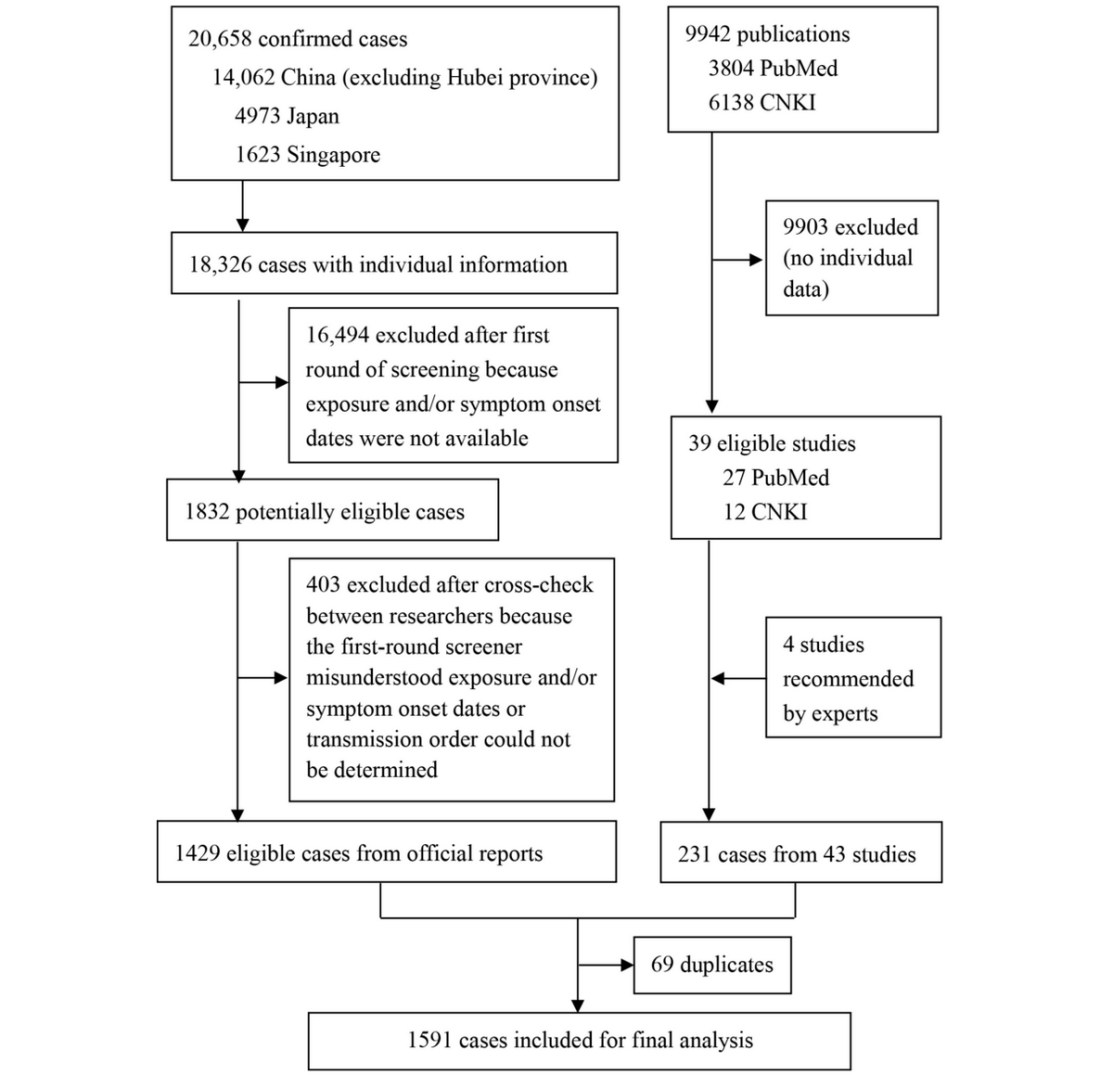
The flowchart of cases selection from the official reports and published studies. CNKI: China National Knowledge Infrastructure.

Figure 1 shows the process of data collection. We systematically retrieved and screened 20,658 case reports and 9942 publications. The 43 publications included in our analysis can be found in Multimedia Appendix 2. In total, 1591 cases were included for the final analysis, including 1211 (76.12%) from China, 301 (18.92%) from Japan, 60 (3.77%) from Singapore, and 19 from five other countries. The cases from China covered 156 cities in 31 provinces of 34 in total.

### Characteristics of Included Cases

The age of included cases ranged from 5 days to 95 years (Table 1). There were 133 cases (10.74%) with a travel history to Hubei province, where Wuhan is the capital city. The source of exposure of 1302 cases (81.99%) was known confirmed cases. The exposure period was exactly 1 day in 508 cases (53.08%), spanned 2-3 days in 227 cases (23.72%), and spanned more than 3 days in the other cases (which were not included in the estimation of parameters involving exposure date). The top 5 initial symptoms were fever (730/1009, 72.35%), cough (257/1009, 25.47%), fatigue (69/1009, 6.84%), sore throat (57/1009, 5.65%), and malaise (54/1009, 5.35%). Clustering cases accounted for 84.92% (1351/1591) of all, and most of them were second generation.

**Table 1:** Characteristics of included cases.

Characteristics		Value	
Age range		5 days to 95 years	
Mean age, years (SD)		45.90 (18.02)	
**Age groups (n=1437), years, n (%)**			
	0-18		89 (6.19)
	19-64		1121 (78.01)
	≥65		227 (15.80)
**Sex (n=** **1559** **), n (%)**			
	Male		768 (49.26)
	Female		791 (50.74)
**Travel history to Hubei (n=1238, n (%)**			
	Yes		133 (10.74)
	No		1105 (89.26)
**Infector was a known confirmed case (n=** **1588** **), n (%)**			
	Yes		1302 (81.99)
	No		286 (18.01)
**Span of exposure period (n=957), n (%)**			
	1 day		508 (53.08)
	2-3 days		227 (23.72)
	>3 days		222 (23.20)
**Top 5 initial symptoms (n=1009), n (%)**			
	Fever		730 (72.35)
	Cough		257 (25.47)
	Fatigue		69 (6.84)
	Sore throat		57 (5.65)
	Malaise		54 (5.35)
**Clustering cases (n=1591), n (%)**			
	Yes		1351 (84.92)
	No		240 (15.08)
**Family cluster (n=1238), n (%)**			
	Yes		514 (41.52)
	No		724 (58.48)
**Generation of clustering cases (n=1350), n (%)**			
	First		61 (4.52)
	Second		1069 (79.19)
	Third		188 (13.93)
	Fourth or higher		32 (2.37)

### Epidemiological Parameters

**Figure 2:**
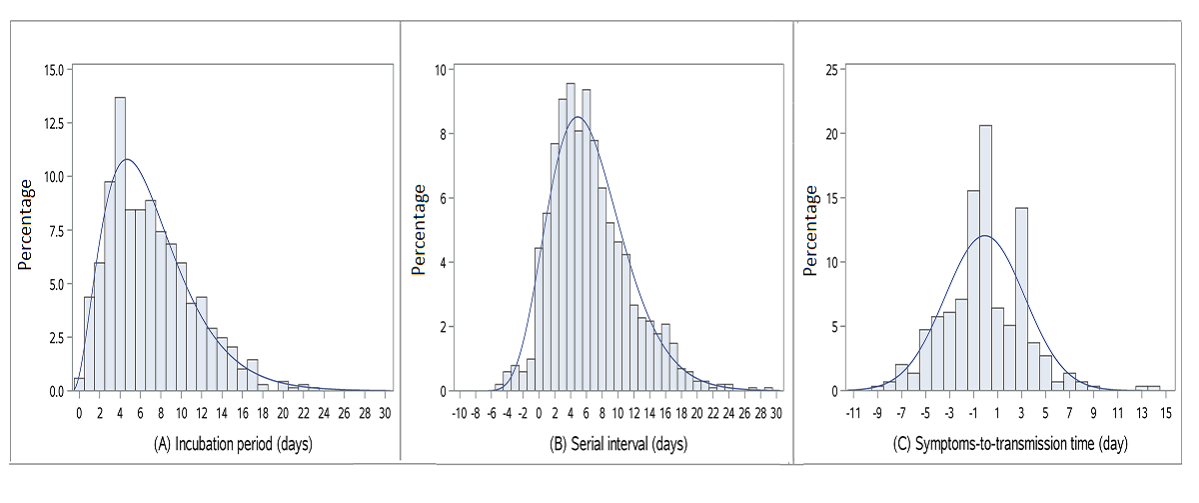
The distribution of (A) incubation period (n=687), (B) serial interval (n=1015 pairs), and (C) symptoms-to-transmission time (n=296 pairs).

Incubation period was estimated from 687 cases, of whom 482 (69.55%) were exposed for 1 day; the remainder were exposed for 2 or 3 days. The incubation periods of individual cases ranged from 0 to 23 days (Table 2), with a median of 6 days; in total, 5.82% (n=40) of incubation periods were longer than 14 days. The incubation period is best described by a gamma distribution (Multimedia Appendix 3, Figure 2A), with a mean of 7.04 days (95% CI 6.74-7.33) and standard deviation of 4.27 days (95% CI 3.92-4.43).

The serial interval was estimated for 1015 infector-infectee pairs and ranged from −5 to 29 days (Table 2), with a median of 6 days. For 32 pairs (3.15%), the serial interval was less than 0; for these 32 pairs, the mean was −2.50 days, meaning that the infectee showed symptoms 2.50 days earlier than the infector. The serial interval is best described by a shifted gamma distribution (Multimedia Appendix 3, Figure 2B), with a mean of 6.49 days (95% CI 6.15-6.80) and standard deviation of 4.90 days (95% CI 4.68-5.25). Based on an exponential growth rate of 0.10 per day [1] and the mean and standard deviation of the serial interval, R_0_ was estimated at 1.85 (95% CI 1.37-2.60). The country-specific R_0_ for China, Japan, and Singapore was 1.83 (95% CI 1.37-2.54), 1.83 (95% CI 1.37-2.53), and 1.98 (95% CI 1.41-2.97), respectively.

The symptoms-to-transmission time was estimated from 296 infector-infectee pairs and ranged from −9 to 14 days (Table 2), with a median of 0 day. Presymptomatic transmission occurred in 129 infector-infectee pairs (43.58%, 95% CI 37.93%-49.23%); for these 129 pairs, the mean symptoms-to-transmission time was −2.88 days, meaning that the transmission occurred 2.88 days before the infectors showed symptoms. Fitted by a normal distribution (Figure 2C), the mean of this parameter was −0.07 days (95% CI −0.43 to 0.31) and the standard deviation was 3.31 days (95% CI 2.96 to 3.66).

The symptoms-to-transmission time can also be inferred by mean serial interval minus mean incubation time (−0.55 days), which is consistent with the direct estimate (−0.07 days) as both suggest the mean symptoms-to-transmission time to be around the day before primary cases’ symptom onset. Based on the above estimates, the timeline of infection for an “average” infector-infectee pair in a transmission chain is demonstrated in Multimedia Appendix 4.

**Table 2:** The estimates of incubation period, serial interval, and symptoms-to-transmission time (unit: day).

Metrics	Incubation period	Serial interval	Symptoms-to-transmission time
Range	0 to 23.00	−5 to 29.00	−9 to 14.00
P_2.5,_ P_97.5_	1.00, 17.00	−1.00, 18.00	−7.00, 7.00
Median (P_25,_ P_75_)	6.00 (4.00, 10.00)	6.00 (3.00, 9.00)	0.00 (−2.00, 2.00)
Mean (95% CI)	7.04 (6.74 to 7.33)	6.49 (6.15 to 6.80)	−0.07 (−0.43 to 0.31)
SD (95% CI)	4.27 (3.92 to 4.43)	4.90 (4.68 to 5.25)	3.31 (2.96 to 3.66)
Mean (95% CI)^a^	6.74 (6.43 to 7.03)	N/A^b^	0.17 (−0.19 to 0.55)
SD (95% CI)^a^	4.34 (3.98 to 4.50)	N/A	3.32 (2.96 to 3.68)

^a^Results of sensitivity analyses. For incubation period, the third day for those whose exposure period spanned three days and the second day for those exposed for two continuous days were used as their exposure dates in sensitivity analyses. For symptoms-to-transmission time, exposure dates were taken in a different way in sensitivity analyses (like those of the incubation period).

^b^N/A: not applicable.

### Sensitivity and Stratified Analyses

In sensitivity analyses, the estimates of incubation period and symptoms-to-transmission time remained stable, with a difference of 0.24-0.3 days from those in primary analysis (Table 2), which represents the largest possible error caused by inclusion of cases with an exposure period spanning 2 or 3 days. The results of the stratified analysis are summarized in Multimedia Appendix 5. The difference between strata was statistically significant in four of the analyses (see footnote of Multimedia Appendix 5). Briefly, the incubation period was longer for those exposed for only 1 day (as compared with those exposed for 2-3 days), while serial interval and symptoms-to-transmission time were longer for those at lower generations of clustered infection. The serial interval was also longer for those infected through nonhousehold contact.

## Discussion

By pooling the individual data of 1591 cases, we estimated the mean incubation period, serial interval, and symptoms-to-transmission time of COVID-19 to be 7.04, 6.49, and −0.07 days, respectively. The incubation period was longer than 14 days in 5.82% of the cases. Infectees’ symptom onsets occurred before those of infectors in 3.15% of the infector-infectee pairs. Presymptomatic transmission occurred in 43.58% of the infector-infectee pairs. R_0_ was estimated to be 1.85 (95% CI 1.37-2.60).

To our knowledge, this study represents the largest case series with accurate data on incubation period, serial interval, and frequency of presymptomatic transmission. Our estimates of incubation period and serial interval are longer than most of the previous estimates [5-10]. There are several possible reasons for the difference. First, the sample size is generally small in previous studies [5,7,9,12-15], but much larger in this one. Second, most cases in previous studies had a long or unclear interval of exposure, making it difficult to determine the exact exposure date [6,8,17] and giving rise to error. By contrast, this study applied strict inclusion criteria regarding the exposure period and a simple method to determine exposure date to ensure the potential error in the estimates was small (<0.3 days, according to the sensitivity analysis). Third, the order of transmission (ie, who is the infector and who is the infectee) in clustering cases, which is crucial to estimation of both parameters, is easy to be mistaken given the possibility of presymptomatic and asymptomatic transmission [21,22,27,28]. According to our experience in screening the publicly available data, for many clusters, there was no clear evidence (eg, who got infected outside and brought the virus to the cluster?) to establish the transmission order. To ensure the accuracy of data, we excluded such clusters in this study, while previous studies rarely described how this issue was handled [11].

Our finding that the incubation period was within 14 days for 94% of the cases lends support to the current practice of 14-day quarantine of persons with potential exposure to SARS-CoV-2. In line with other studies [17], we also found some cases who developed symptoms more than 14 days after exposure, indicating that longer quarantine periods might be justified for some people. However, as it is hard to know beforehand who will develop symptoms beyond 14 days after exposure, the cost of extending mandatory quarantine of many people and the potential consequence of failure to identify a few symptomatic cases must be weighed carefully. Testing at the end of quarantine and social distancing afterwards may help reduce the risk associated with this phenomenon.

The negative values of the serial interval and symptoms-to-transmission time provide evidence of presymptomatic transmission. Specifically, 43.58% of the transmission events in this study occurred before infectors’ symptom onsets. Asymptomatic transmission was also reported by the publications included in this study (Multimedia Appendix 2). These phenomena constitute a challenge in the control of the epidemic and highlight the importance of adequate testing, strict quarantine, and social distancing to reduce the transmission caused by “hidden” cases.

The R_0_ we estimated is smaller than those from previous estimates, which were mostly between 2 and 4 [20,29,30]. The difference may be due to either the methodological issues in obtaining parameters as discussed above or due to the estimating method itself. In estimating R_0_, the serial interval was used to approximate generation time in the Euler-Lotka equation, which may lead to underestimation because of the possibly differing incubation periods of the infector and infectee. Assuming the incubation periods of the infector and infectee follow the same distribution, we found that the R_0_ was 1.90, almost the same as the original one, suggesting a small error in our estimation. In any case, a smaller R_0_ should not be interpreted as a low risk of transmission. Slow response of government, presymptomatic and asymptomatic transmission, and insufficient protection measures taken by the public together could lead to an out-of-control epidemic, as is the current situation in many countries.

This study has some limitations. First, it was based on publicly reported cases. Previous studies suggested that such cases may overrepresent the severe ones. However, as the publicly available information on severity was incomplete and inaccurate, we were unable to assess the magnitude and potential impact of this issue. Another problem with publicly reported cases is that their epidemiology history might be reported in more detail at the early stage of epidemic than when the number of cases grew larger. We found that this was especially true for Japan and Singapore. Thus, in general, the results of this study reflected more the situation during the early stage of the pandemic. Second, to ensure accuracy in estimating the incubation period, the cases exposed for a long (>3 days) or unclear period were excluded (which was inevitable and justifiable). This might have contributed partly to the longer incubation period in this study than others, as there was a trend toward a longer incubation period in the cases exposed for 1 day only (a relatively shorter period) as compared with those exposed for 2-3 days (Multimedia Appendix 5). Third, R_0_ might have been underestimated as the Euler-Lotka equation was used; however, as explained in the last paragraph, the magnitude of underestimation, if any, was very small.

In conclusion, this study obtained robust estimates of several key epidemiological parameters of COVID-19. It provides additional evidence on the mean incubation period of COVID-19, which supports the current practice of 14-day quarantine of persons with potential exposure but also suggests the need for additional measures. Presymptomatic transmission together with the asymptomatic transmission reported by previous studies highlight the importance of adequate testing, strict quarantine, and social distancing.

## Appendix

Multimedia Appendix 1 A full list of the data sources (except academic studies).

Multimedia Appendix 2 A list of eligible studies included for analysis.

Multimedia Appendix 3 The Akaike information criterion scores and shifted terms for lognormal, Weibull, and Gamma distributions.

Multimedia Appendix 4 The timeline of events for an "average" infector-infectee pair in a transmission chain, according to the estimates from this study. E1: exposure of infector; E2: exposure of infectee; O1: symptom onset of infector; O2: symptom onset of infectee.

Multimedia Appendix 5 Stratified analyses of incubation period, serial interval, and symptoms-to-transmission time according to the selected characteristic.
