# Supplementary material for: Epidemiological Parameters of COVID-19: Case Series Study
Source: J Med Internet Res. 2020 Oct 12;22(10):e19994. doi: 10.2196/19994 (PMC7553786; doi:10.2196/19994)
Supplement: Multimedia Appendix 2 [file jmir_v22i10e19994_app2.docx]

**Appendix 2. A list of eligible studies included for analysis**

1. Rothe, C.*, et al.* Transmission of 2019-nCoV Infection from an Asymptomatic Contact in Germany. *The New England journal of medicine* **382**, 970-971 (2020).

2. Bai, Y.*, et al.* Presumed Asymptomatic Carrier Transmission of COVID-19. *Jama*, <http://doi.org/10.1001/jama.2020.2565> (2020).

3. Tong, Z.D.*, et al.* Potential Presymptomatic Transmission of SARS-CoV-2, Zhejiang Province, China, 2020. *Emerging infectious diseases* **26**(2020).

4. Liu, Y.C., Liao, C.H., Chang, C.F., Chou, C.C. & Lin, Y.R. A Locally Transmitted Case of SARS-CoV-2 Infection in Taiwan. *The New England journal of medicine* **382**, 1070-1072 (2020).

5. Wang, K.S., Ye, Z.H. & Zhang, D. Investigation on a cluster of coronavirus disease 2019 in Pingyang County. *Preventive Medicine*, 1-6 (2020).

6. Huang, R., Xia, J., Chen, Y., Shan, C. & Wu, C. A family cluster of SARS-CoV-2 infection involving 11 patients in Nanjing, China. *The Lancet. Infectious diseases*, <http://doi.org/10.1016/s1473-3099(1020)30147-x> (2020).

7. Liu, M.*, et al.* Family cluster of child SARS⁃CoV⁃2 infections: a case report. *Medical Journal of Wuhan University*, 1-5 (2020).

8. Chan, J.F.*, et al.* A familial cluster of pneumonia associated with the 2019 novel coronavirus indicating person-to-person transmission: a study of a family cluster. *Lancet* **395**, 514-523 (2020).

9. Yu, P., Zhu, J., Zhang, Z., Han, Y. & Huang, L. A familial cluster of infection associated with the 2019 novel coronavirus indicating potential person-to-person transmission during the incubation period. *The Journal of infectious diseases*, <http://doi.org/10.1093/infdis/jiaa1077> (2020).

10. Zhou, H.*, et al.* Epidemiological survey on a family aggregation COVID-19 in Y County, Chenzhou City, Hunan. *Shanghai Journal of Preventive Medicine* **32**, 1-4 (2020).

11. Guan, Q.*, et al.* Epidemiological survey on a family aggregation COVID-19. *Chinese Journal of Epidemiology* **5**, 629-633 (2020).

12. Luo, K.W.*, et al.* An epidemiological investigation of 2019 novel coronavirus diseases through aerosol-borne transmission by public transport. *Practical Preventive Medicine*, 1-3 (2020).

13. Li, Y.G.*, et al.* An epidemiological investigation of 2019 novel coronavirus (2019-nCoV) disease in a department store in Tianjin. *Chinese Journal of Epidemiology* **41**, 489-493 (2020).

14. Ki, M. & nCo, V.T. Epidemiologic characteristics of early cases with 2019 novel coronavirus (2019-nCoV) disease in Republic of Korea. *Epidemiology and health*, e2020007, <http://doi.org/2020010.2024178/epih.e2020007> (2020).

15. Li, Q.*, et al.* Early Transmission Dynamics in Wuhan, China, of Novel Coronavirus-Infected Pneumonia. *The New England journal of medicine*, <http://doi.org/10.1056/NEJMoa2001316> (2020).

16. Sun, Q.L.*, et al.* A clustering epidemic of pneumonia caused by 2019-nCoV. *Practical Preventive Medicine*, 1-4 (2020).

17. Du, Y.H.*, et al.* Clinical features and CT signs of early family clustering novel coronavirus pneumonia. *Journal of Xi’an Jiaotong University (Medical Sciences)*, 1-7 (2020).

18. Bai, S.L.*, et al.* Analysis of the first cluster of cases in a family of novel coronavirus pneumonia in Gansu Province. *Zhonghua yu fang yi xue za zhi [Chinese journal of preventive medicine]* **54**, E005 (2020).

19. Kam, K.Q.*, et al.* A Well Infant with Coronavirus Disease 2019 (COVID-19) with High Viral Load. *Clinical infectious diseases,* <http://doi.org/10.1093/cid/ciaa1201> (2020).

20. Zhang, G.X.*, et al.* Twin girls infected with SARS-CoV-2. *Zhongguo dang dai er ke za zhi = Chinese journal of contemporary pediatrics* **22**, 221-225 (2020).

21. Ge, R.*, et al.* The role of close contacts tracking management in COVID-19 prevention: A cluster investigation in Jiaxing, China. *The Journal of infection*, <http://doi.org/10.1016/j.jinf.2020.1003.1015> (2020).

22. Chen, D.*, et al.* Recurrence of positive SARS-CoV-2 RNA in COVID-19: A case report. *International journal of infectious diseases* **93**, 297-299 (2020).

23. Wen, R., Sun, Y. & Xing, Q.S. A patient with SARS-CoV-2 infection during pregnancy in Qingdao, China. *Journal of microbiology, immunology, and infection = Wei mian yu gan ran za zhi*, <http://doi.org/10.1016/j.jmii.2020.1003.1004> (2020).

24. Xiao, W.J.*, et al.* Investigation of an epidemic cluster caused by COVID rectangle19 cases in incubation period in Shanghai. *Zhonghua liu xing bing xue za zhi* **41**, E033 (2020).

25. liu, Z.*, et al.* Investigation of a COVID-19 case with unknown cause and its close contacts. *Journal of Shandong University (Health Sciences)* **58**, 1-5 (2020).

26. Park, J.Y., Han, M.S., Park, K.U., Kim, J.Y. & Choi, E.H. First Pediatric Case of Coronavirus Disease 2019 in Korea. *Journal of Korean medical science* **35**, e124 (2020).

27. Ghinai, I.*, et al.* First known person-to-person transmission of severe acute respiratory syndrome coronavirus 2 (SARS-CoV-2) in the USA. *Lancet* **395**, 1137-1144 (2020).

28. Sibylle, B.S.*, et al.* First cases of coronavirus disease 2019 (COVID-19) in France: surveillance, investigations and control measures, January 2020. *Euro surveillance* **25**(2020).

29. Alonso Diaz, C., Lopez Maestro, M., Moral Pumarega, M.T., Flores Anton, B. & Pallas Alonso, C. First case of neonatal infection due to SARS-CoV-2 in Spain. *Anales de pediatria*, <http://doi.org/10.1016/j.anpedi.2020.1003.1002> (2020).

30. Zhang, J., Tian, S., Lou, J. & Chen, Y. Familial cluster of COVID-19 infection from an asymptomatic. *Critical care* **24**, 119 (2020).

31. Zhang, Y.*, et al.* Epidemiological investigation on a cluster epidemic of COVID-19 in a collective workplace in Tianjin. *Zhonghua liu xing bing xue za zhi* **41**, 649-653 (2020).

32. Chang, C.Y.*, et al.* Epidemiological characteristics of the first COVID-19 case and related family cluster outbreaks in Jinan. *Journal of Shandong University (Health Sciences)* **58**, 1-5 (2020).

33. Qiu, Y.Y.*, et al.* Epidemiological analysis on a family cluster of COVID-19. *Zhonghua liu xing bing xue za zhi* **41**, 506-509 (2020).

34. Ye, F.*, et al.* Delivery of infection from asymptomatic carriers of COVID-19 in a familial cluster. *International journal of infectious diseases*, <http://doi.org/10.1016/j.ijid.2020.1003.1042> (2020).

35. Qian, G.*, et al.* A COVID-19 Transmission within a family cluster by presymptomatic infectors in China. *Clinical infectious diseases*, <http://doi.org/10.1093/cid/ciaa1316> (2020).

36. Huang, J.*, et al.* COVID-19 in post-transplantation patients- report of two cases. *American journal of transplantation*, <http://doi.org/10.1111/ajt.15896> (2020).

37. Li, C.X., Wu, B., Luo, F. & Zhang, N. Clinical Study and CT Findings of a Familial Cluster of Pneumonia with Coronavirus Disease 2019 (COVID-19). *Journal of Sichuan University. Medical science edition* **51**, 155-158 (2020).

38. Li, J.M., Yi, Q., Wei, M.G., Wei, H.L. & Yuan, S.S. Clinical management and epidemiological measures to a second-generation patient with COVID-19 and her close contacts: A case report. *Acad J Chin PLA Med Sch* **41**, 1-3 (2020).

39. Feng, H., Liu, Y., Lv, M. & Zhong, J. A case report of COVID-19 with false negative RT-PCR test: necessity of chest CT. *Japanese journal of radiology*, <http://doi.org/10.1007/s11604-11020-00967-11609> (2020).

40. Li, C.*, et al.* Asymptomatic and Human-to-Human Transmission of SARS-CoV-2 in a 2-Family Cluster, Xuzhou, China. *Emerging infectious diseases* **26**(2020).

41. Mi, J.*, et al.* Analysis of cluster epidemic of corona virus disease 2019. *J Bengbu Med Coll* **45**, 147-149 (2020).

42. Wang, W.R.*, et al.* Analysis of a family cluster outbreak of coronavirus disease 2019 in Jinan. *Journal of Shandong University (Health Sciences)* **58**, 1-5 (2020).

43. Lu, S.*, et al.* Alert for non-respiratory symptoms of Coronavirus Disease 2019 (COVID-19) patients in epidemic period: A case report of familial cluster with three asymptomatic COVID-19 patients. *Journal of medical virology*, <http://doi.org/10.1002/jmv.25776> (2020).
