# Supplementary figures and images for: Epidemiological Parameters of COVID-19: Case Series Study
Source: J Med Internet Res. 2020 Oct 12;22(10):e19994. doi: 10.2196/19994 (PMC7553786; doi:10.2196/19994)

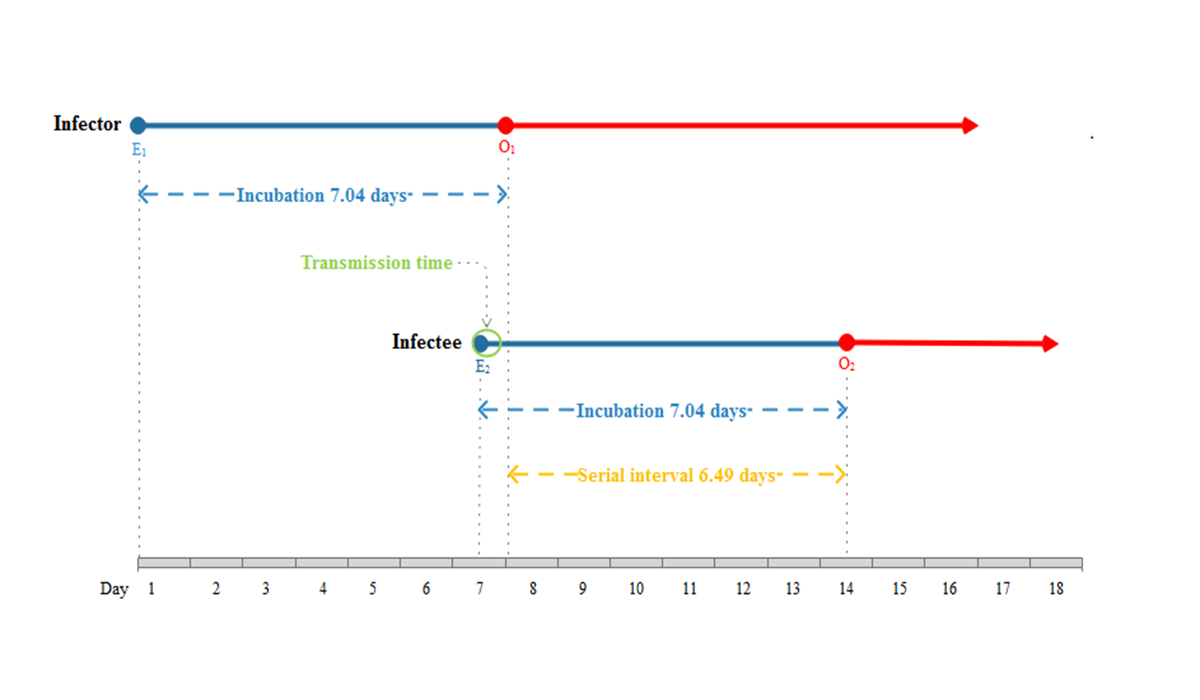

Supplement: Multimedia Appendix 4 [file jmir_v22i10e19994_app4.png]
