# Supplementary material for: Epidemiological Parameters of COVID-19: Case Series Study
Source: J Med Internet Res. 2020 Oct 12;22(10):e19994. doi: 10.2196/19994 (PMC7553786; doi:10.2196/19994)
Supplement: Multimedia Appendix 5 [file jmir_v22i10e19994_app5.docx]

Appendix 5. Stratified analyses of incubation period, serial interval, and symptoms-to-transmission time according to selected characteristics

| Groups | Incubation period | Serial interval | Symptoms-to-transmission time |
| --- | --- | --- | --- |
| **Age (year)** |  |  |  |
| 0 - 18 | 7.19 (5.21, 9.24) | -- | -- |
| 19 - 64 | 6.96 (6.62, 7.31) | -- | -- |
| ≥ 65 | 8.89 (7.30, 9.84) | -- | -- |
| **Sex** |  |  |  |
| Male | 6.76 (6.34, 7.19) | -- | -- |
| Female | 7.55 (7.06, 8.07) | -- | -- |
| **Travel history to Hubei** |  |  |  |
| Yes | 7.20 (6.46, 7.94) | -- | -- |
| No | 7.00 (6.62, 7.36) | -- | -- |
| **Infector was a known confirmed case origin** |  |  |  |
| Yes | 7.12 (6.73, 7.52) | -- | -- |
| No | 6.95 (6.49, 7.41) | -- | -- |
| **Span of exposure period** |  |  |  |
| 1 day | 7.31 (6.94, 7.72)* | -- | 0.01 (−0.42, 0.48) |
| 2-3 days | 6.41 (5.84, 6.92)* | -- | −0.32 (−1.01, 0.46) |
| **Household contact** |  |  |  |
| Yes | 6.16 (4.68, 7.74) | 5.85 (5.37, 6.28)* | 0.24 (−0.64, 1.17) |
| No | 7.09 (6.76, 7.43) | 7.00 (6.42, 7.63)* | −0.06 (−0.46, 0.34) |
| **Clustering cases** |  |  |  |
| Yes | 6.92 (6.54, 7.31) | 6.51 (6.20, 6.80) | -0.03 (-0.39, 0.35) |
| No | 7.28 (6.71, 7.86) | -- | -- |
| **Generation of clustering cases** |  |  |  |
| 1^st^ | 5.67 (4.97, 6.46) | -- | -- |
| 2^nd^ | 7.33 (6.86, 7.75) | 6.81 (6.44, 7.16)* | 0.23 (−0.18, 0.66)* |
| 3^rd^ or higher | 5.98 (5.08, 7.04) | 5.14 (4.58, 5.76)* | −1.17 (−1.89, −0.30)* |

*The difference between strata was statistically significant (*P*<.001). --, not applicable, or not available because of too few cases.
